# Supplementary material for: Quantification of the pace of biological aging in humans through a blood test, the DunedinPoAm DNA methylation algorithm
Source: eLife. 2020 May 5;9:e54870. doi: 10.7554/eLife.54870 (PMC7282814; doi:10.7554/eLife.54870)
Supplement: Supplementary file 1. — Tables include (1) a list of the CpGs included in the DunedinPoAm algorithm and their associated weights, (2-7) results from regression models reported in the main text, and (8) a description of the functional test measures from the Dunedin Study. Supplementary figures show (1) analysis of the Dunedin Study to develop Dunedin PoAm and (1-6) effect-sizes for associations of DunedinPoAm and the epigenetic clocks porposed by Horvath, Hannum et al., and Levine et al. with outcome measures. [file elife-54870-supp1.docx]

­­­­**Supplementary File 1A. Probes and associated weights composing the DunedinPoAm algorithm.** The DunedinPoAm algorithm is a linear combination of 46 CpG methylation beta values weighted by coefficients estimated in the elastic net regression and added to the model intercept value of -0.06. (For sensitivity analyses addressing normalization method, and specific probes, see Supplement section 3.) R code to implement the DunedinPoAm scoring algorithm is included as supplemental file “DunedinPoAm_Projector.zip”.

­­­­**Supplementary File 1B. Comparison of age-38 DundedinPoAm and age 26-38 Pace of Aging effect-sizes for analysis of healthspan-related characteristics.** The table shows effect-sizes for analysis of healthspan-related characteristics at age 45 years. mPoA was measured from blood DNA methylation collected when Study members were aged 38 years. Pace of Aging was measured from longitudinal change in 18 biomarkers across measurements made at ages 26, 32, and 38 years. Sample restricted to N=810 Study members with data on mPoA and Pace of Aging. Effect-sizes correspond to the analysis reported in **Supplemental Figure 1B** and are reported in terms of standard deviation differences in the age-45 outcome associated with a 1 standard deviation increase in mPoA (i.e. effect-sizes are interpretable as Pearson r). All models were adjusted for sex.

­­­­**Supplementary File 1C. Effect-sizes for associations of age-38 DundedinPoAm and epigenetic clocks with healthspan-related characteristics at age 45 and change in healthspan characteristics from age 38-45.** Panel A of the table shows effect-sizes for analysis of healthspan-related characteristics at age 45 years. Panel B of the table shows effect-sizes for analysis of change in healthspan-related characteristics from 38 to 45. Methylation measurements were derived from blood DNA methylation collected when Study members were aged 38 years. Prior to analysis, Horvath, Hannum, and Levine Clock values were residualized for chronological age. Effect-sizes are reported in terms of standard deviation differences in the outcome associated with a 1 standard deviation increase in methylation measures (i.e. effect-sizes are interpretable as Pearson r). All models were adjusted for sex.

**Panel A. Analysis of healthspan-related characteristics at age 45 years**

**Panel B. Analysis of change in healthspan-related characteristics from age 38-45 years**

**Supplementary File 1D. Effect-sizes for associations of DunedinPoAm and epigenetic clocks with KDM Biological Age, and self-rated health in the Understanding Society Study.** The table reports standardized regression coefficients and their standard errors from linear regression models in which the predictor was the methylation measure listed in the far-left column and the dependent variable was either KDM Biological Age (left side coefficients) or self-rated health (right side coefficients). All models included sex and chronological age as covariates. Model 2 included covariates for cell counts estimated from the methylation data. Model 3 included covariates adjusting for smoking status. Model 4 included nonsmokers only. Prior to analysis, Horvath, Hannum, and Levine Clock values were residualized for chronological age. All methylation variables were residualized for plate number.

**Supplementary File 1E. Effect-sizes for associations of DunedinPoAm and epigenetic clocks with morbidity and mortality in the Normative Aging Study.** Time-to-event analyses of mortality and chronic disease incidence (Panels A and B) were conducted using Cox proportional hazard models to estimate hazard ratios (HRs) in N=771 participants. Repeated-measures analysis (Panel C) was conducted using Poisson regression within a generalized estimating equations framework to account for the nonindependence of repeated observations of individuals (N=1,488 observations of 771 individuals). All models included covariate adjustment for chronological age. Smoking status was measured from the American Thoracic Society Questionnaire (Ferris, 1978) completed by participants at each assessment wave. We classified participants as being current, former, or never smokers (Gao et al., 2019a). Pack years is the total number of cigarettes smoked across the participants lifetime in units equivalent to the number of cigarettes smoked during a year of smoking 1 pack (20 cigarettes) per day. Age acceleration residuals for epigenetic clocks were calculated by regressing epigenetic age on chronological age and predicting residual values. All methylation measures were standardized to M=0 SD=1 for analysis. Effect-sizes thus reflect risk associated with a 1-SD increase in the methylation measure.

**Supplementary File 1F. Effect-sizes for associations of socioeconomic status (SES) and victimization exposure with DunedinPoAm and epigenetic clocks at age 18 in the E-Risk Study.** The table shows effect-sizes reported as standardized regression coefficients (b) and 95% confidence intervals (CIs) from models in which childhood family socioeconomic status (SES) and victimization were predictor variables and the dependent variables were the DNA methylation measures. Model 1 included covariate adjustment for sex. Model 2 additionally included covariates for estimated cell counts. (Chronological age was the same for all twins in the birth cohort.) Models 3 and 4 adjusted for smoking status. Only 33% of the analysis sample had ever smoked. Model 3 included covariates adjusting for smoking status at age 18 (never, former, current). Model 4 included nonsmokers only. Methylation measurements were derived from blood DNA methylation collected when Study members were aged 18 years and were residualized for plate number prior to analysis. All E-Risk participants are the same chronological age. Epigenetic clock measures were differenced from this chronological age prior to analysis. Effect-sizes are reported in terms of standard deviation differences in the outcome associated with a 1 standard deviation increase in methylation measures. All models were adjusted for sex. Standard errors were clustered at the family level to account for non-independence of twin data.

**Supplementary File 1G. Characteristics of participants in the CALERIE Trial and subsample with baseline methylation data.** The top panel of the table shows demographic characteristics and measured rate of aging for participants in the Ad Libitum (usual diet) and Caloric Restriction arms of the CALERIE Trial. The middle panel shows the same data for the subset of participants for whom methylation data were available from the baseline CALERIE assessment. The bottom panel shows effect-sizes for associations of methylation measures with the rate of change in KDM Biological Age during follow-up. Effect-sizes are stratified marginal effects computed from regressions of predicted slopes of change in KDM Biological Age on treatment condition, baseline values of the methylation measures, and the interaction of treatment condition and the methylation measures. Effect-sizes for association of baseline DunedinPoAm with rate of change in KDM Biological Age over follow-up are plotted separately by treatment condition (CR for caloric restriction and AL for Ad Libitum control). Effect-sizes reflect the predicted increase in the rate of annual change in KDM Biological Age over the 2 years of follow-up associated with a 1 SD increase in the methylation measure. For example, for DunedinPoaM, a value of 0.2 for participants in the AL control condition indicates that having DunedinPoAm 1 SD higher at baseline is associated with an increase in the aging rate of 0.2 years of physiological change per 12 months of follow-up. Models included covariate adjustment for sex and chronological age at baseline.

**Effect-sizes for associations of DunedinPoAm and epigenetic clocks with future rate of change in KDM Biological Age in Ad Libitum (AL) control group and Caloric Restriction (CR) intervention group participants.**

**Supplementary File 1H. Physical and cognitive functioning and subjective signs of aging measures in the Dunedin Study**

| **Physical Functioning** (N=800 with DunedinPoAm data) | |
| --- | --- |
| Balance | Balance was measured using the Unipedal Stance Test as the maximum time achieved across three trials of the test with eyes closed (Bohannon et al., 1984; Springer et al., 2007; Vereeck et al., 2008). |
| Gait Speed | Gait speed (meters per second) was assessed with the 6-m-long GAITRite Electronic Walkway (CIR Systems, Inc) with 2-m acceleration and 2-m deceleration before and after the walkway, respectively. Gait speed was assessed under 3 walking conditions: usual gait speed (walk at normal pace from a standing start, measured as a mean of 2 walks) and 2 challenge paradigms, dual-task gait speed (walk at normal pace while reciting alternate letters of the alphabet out loud, starting with the letter “A,” measured as a mean of 2 walks) and maximum gait speed (walk as fast as safely possible, measured as a mean of 3 walks). We calculated the mean of the 3 individual walk conditions to generate our primary measure of composite gait speed (Rasmussen et al., 2019). |
| Steps in Place | The 2-min step test was measured as the number of times a participant lifted their right knee to mid-thigh height (measured as the height half-way between the knee cap and the iliac crest) in 2 minutes at a self-directed pace (Jones and Rikli, 2002; Rikli and Jones, 1999). |
| Chair Stands | Chair rises were measured as the number of stands a participant completed in 30 seconds from a seated position (Jones et al., 1999; Jones and Rikli, 2002). |
| Grip Strength | Handgrip strength was measured for the dominant hand (elbow held at 90°, upper arm held tight against the trunk) as the maximum value achieved across three trials using a Jamar digital dynamometer (Mathiowetz et al., 1985; Rantanen T et al., 1999). |
| Motor Coordination | At ages 38 and 45, we measured motor functioning as the time to completion of the Grooved Pegboard Test with the dominant hand. |
| Physical Limitations | Physical limitations were measured with the 10-item RAND 36-Item Health Survey 1.0 physical functioning scale (Ware and Sherbourne, 1992). Participant responses (“limited a lot”, “limited a little”, “not limited at all”) assessed their difficulty with completing various activities, e.g., climbing several flights of stairs, walking more than 1 km, participating in strenuous sports, etc. Scores were reversed to reflect physical limitations so that a high score indicates more limitations. |
| Decline in Physical Functioning | Tests of balance and grip strength and interviews about physical limitations were completed at both the age-38 and age-45 Dunedin Study assessments. We measured decline across the 7-year measurement interval by subtracting the age-38 test score from the age-45 test score. |
| **Cognitive Functioning** (N=795 with mPoA data) | |
| Cognitive Functioning | The Wechsler Adult Intelligence Scale-IV (WAIS-IV) (Wechsler, 2008) was administered to the participants at age 45 years, yielding the IQ. In addition to full scale IQ, the WAIS-IV measures four specific domains of cognitive function: Processing Speed, Working Memory, Perceptual Reasoning, and Verbal Comprehension. |
| Cognitive Decline | IQ is a highly reliable measure of general intellectual functioning that captures overall ability across differentiable cognitive functions. We measured IQ from the individually administered Wechsler Intelligence Scale for Children-Revised (WISC-R; averaged across ages 7, 9, 11, and 13)(Wechsler, 2003) and the Wechsler Adult Intelligence Scale-IV (WAIS-IV; age 45) (Wechsler, 2008). We measured IQ decline by comparing scores from the WISC-R and the WAIS-IV. |
| **Subjective Signs of Aging** (N=802 with mPoA data) | |
| Self-rated Health | Study members rated their health on a scale of 1-5 (poor, fair, good, very good, or excellent). |
| Facial Aging | Facial Aging is the subjective perception of aged appearance based on a facial photograph and is proposed as a clinically-useful marker of mortality risk (Christensen et al., 2009). Facial Aging measurement in the Dunedin Study was based on ratings by an independent panel of 8 raters of each participant’s facial photograph (Belsky et al., 2015; Shalev et al., 2014). Facial Aging was based on two measurements of perceived age. First, Age Range was assessed by an independent panel of 4 raters, who were presented with standardized (non-smiling) facial photographs of participants and were kept blind to their actual age. Raters used a Likert scale to categorize each participant into a 5-year age range (i.e., from 20-24 years old up to 70+ years old) (interrater reliability = .77). Scores for each participant were averaged across all raters. Second, Relative Age was assessed by a different panel of 4 raters, who were told that all photos were of people aged 45 years old. Raters then used a 7-item Likert scale to assign a “relative age” to each participant (1=“young looking”, 7=“old looking”) (interrater reliability = .79). The measure of perceived age at 45 years, Facial Age, was derived by standardizing and averaging Age Range and Relative Age scores. |
| Subjective Decline | Self-rated Health and Facial Aging were measured at both the age-38 and age-45 assessments. We measured decline in self-rated health as incident fair/poor health reported at the age-45 assessment. We measured acceleration in Facial Aging by computing the difference in Facial Aging Z-scores between the age-45 and age-38 assessments. |
